# Revolutionizing cancer treatment: the power of bi- and tri-specific T-cell engagers in oncolytic virotherapy

**DOI:** 10.3389/fimmu.2024.1343378

**Published:** 2024-02-22

**Authors:** Ali Zarezadeh Mehrabadi, Mahdi Tat, Akbar Ghorbani Alvanegh, Fatemeh Roozbahani, Hadi Esmaeili Gouvarchin Ghaleh

**Affiliations:** ^1^ Applied Virology Research Center, Baqiyatallah University of Medical Sciences, Tehran, Iran; ^2^ Human Genetics Research Center, Baqiyatallah University of Medical Sciences, Tehran, Iran; ^3^ Department of Microbiology and Virology, Faculty of Medicine, Mazandaran University of Medical Sciences, Sari, Iran

**Keywords:** cancer immunotherapy, oncolytic viruses, bi-specific t cell engagers, tri-specific t cell engagers, combination therapy

## Abstract

Bi- or tri-specific T cell engagers (BiTE or TriTE) are recombinant bispecific proteins designed to stimulate T-cell immunity directly, bypassing antigen presentation by antigen-presenting cells (APCs). However, these molecules suffer from limitations such as short biological half-life and poor residence time in the tumor microenvironment (TME). Fortunately, these challenges can be overcome when combined with OVs. Various strategies have been developed, such as encoding secretory BiTEs within OV vectors, resulting in improved targeting and activation of T cells, secretion of key cytokines, and bystander killing of tumor cells. Additionally, oncolytic viruses armed with BiTEs have shown promising outcomes in enhancing major histocompatibility complex I antigen (MHC-I) presentation, T-cell proliferation, activation, and cytotoxicity against tumor cells. These combined approaches address tumor heterogeneity, drug delivery, and T-cell infiltration, offering a comprehensive and effective solution. This review article aims to provide a comprehensive overview of Bi- or TriTEs and OVs as promising therapeutic approaches in the field of cancer treatment. We summarize the cutting-edge advancements in oncolytic virotherapy immune-related genetic engineering, focusing on the innovative combination of BiTE or TriTE with OVs.

## 1 Introduction

Cancer is one of the most significant public health issues globally (1). According to the GLOBOCAN 2020 study, the estimated number of cancer cases worldwide in 2020 exceeded 19 million patients, while the number of cancer-related deaths approached about ten million cases (2). Therefore, developing an efficient health system to improve preventive and therapeutic interventions is imperative for dealing with this challenge.

To date, a range of therapeutic approaches have been developed for managing malignancies. Surgery is widely recognized as an essential and prevalent treatment for solid tumors, although accompanied by numerous risks such as cancer metastasis (3, 4). Alongside surgery, chemotherapy and radiotherapy represent two prominent procedures employed in cancer treatment. Despite their unavoidable benefits, these approaches are not successful in eradicating tumors in many cases (5, 6). Therefore, novel and less complicated cancer therapies such as monoclonal antibodies have become developed, particularly due to the systemic adverse effects of traditional treatments on healthy tissues and organs (2, 7). Bi- and Tri-specific T cell engagers (BiTEs and TriTEs) as well as OVs are two innovative therapeutic approaches that are currently the subject of numerous ongoing clinical trials due to their promising therapeutic potential (8–10).

OVs-mediated immunotherapy exhibits a targeted strategy by specifically targeting cancer cells, infecting and lysing them, while refraining from infecting not malignant cells. The OVs encompass both wild type viruses and genetically engineered variants derived from wild viruses (11). Furthermore, beyond to their oncolytic activities, OVs have demonstrated considerable efficacy in inducing inflammation and triggering immune responses against both the viruses and the tumor cells. Nevertheless, the immune response’s outcome is accompanied by some complications; the anti-tumor immunity facilitated by OVs mediated cancer immunotherapy eventually appears to be efficient (12, 13).

OVs serve as an appropriate platform for the delivery of therapeutic genes, facilitating the development of different mechanisms of action (14, 15). There are several categories of Trans genes that can be integrated to OV vectors. These genes have the potential to produce cytokines that induce cellular immunity, such as IL-2, IL-12, and IL-15 (16–18). Furthermore, genes involved in the production of proteins that trigger apoptosis and necrosis in malignant cells, such as TRAIL and TNF-α, are also employed in the development of engineered OVs (18, 19). In addition to these genes that have been applied in preclinical and clinical studies, there has been a new focus on genes encoding antibodies with the ability to identify immune cell-associated antigens and tumor-associated antigens that are readily accessible. These therapeutic approaches known as BiTEs and TriTEs which are considered as an innovative class of immunotherapeutic agents (20).

BiTE is a recombinant bispecific antibody with two linked single-chain fragment variables (scFvs) derived from separate antibodies, one targeting a specific cell-surface molecule on T cells while the other scFv targets antigens present on the surface of cancer cells (21). TriTEs are capable of identifying three distinct targeted antigens. A heterologous scFv employed to recognize one or two tumor antigens, which then be linked to another scFv specify for T or NK cell antigens (22).

In this review, our primary focus lies on the application of OVs as a vector to produce bi- or tri-specific antibodies that are facilitate interactions between tumor cells and T or NK cells. This interaction ultimately leads to the activation of immune cells and triggering of tumoricidal activity. Recent findings in pre-clinical and clinical studies involving OVs armed with various BiTEs and TriTEs antibodies for cancer immunotherapy will be discussed.

## 2 Overview of the bi- and tri-specific T cell engagers and cancer immunotherapy

The concept of using molecules with multiple binding sites for improving their biological functionality back to early 60s, when first bispecific molecule were developed through a combination of antigen-binding fragments derived from distinct polyclonal sera (23). The production of bispecific antibodies was significant progress in the 1970s and 1980s by the advancement of chemical conjugation methods for combining two distinct antigen-specific monoclonal antibodies, as well as the fusing of hybridoma cell lines (quadromas) that are suitable for producing these molecules (24–26). Nevertheless, early formats of these recombinant proteins had restricted therapeutic effectiveness, with advancements in genetic engineering, there are already More than one hundred polyspecific antibody formats under clinical evaluation (27, 28). Although the initial focus was placed on hematological malignancies, there are ongoing researches for the treatment of solid tumors.

**Figure 1:**
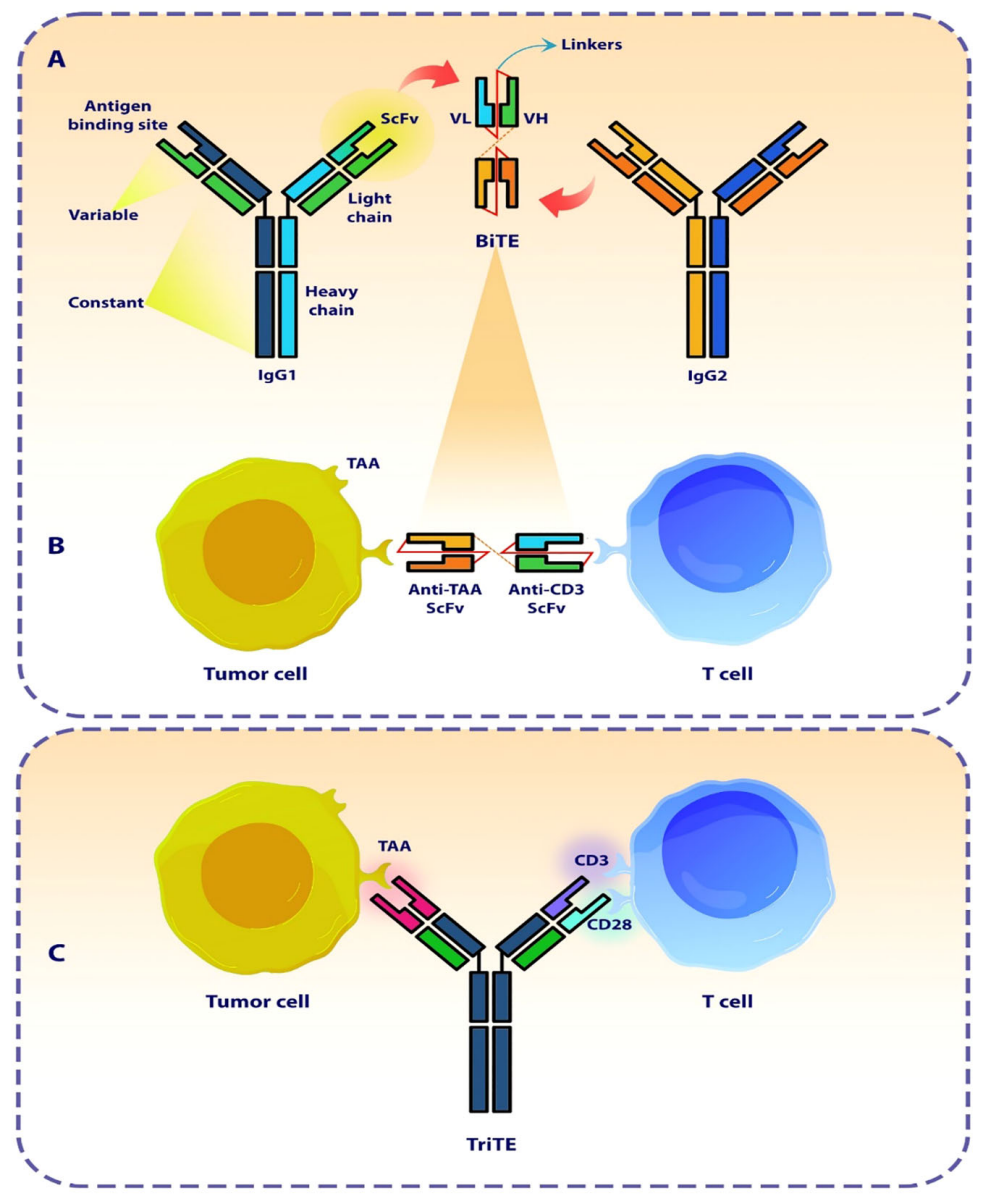
The design of a bi- and tri-specific T cell engager antibody (BiTEs and TriTE). **(A)** The Schematic represents the structure and origin of a BiTE molecule that are derived from two distinct antibodies, one specific for a T cell activation molecule and the other specific for a TAA. **(B)** The BiTE molecule organizes the formation of an immunologic synapse by concurrently interacting with a tumor cell via TAA and a T cell through CD3. **(C)** The Schematic represents the conceptualization and design of a TriTE antibody, demonstrating its mechanism of action in establishing a link between T cells and cancer cells. SCFV, single chain fragment variant; VH, Heavy chain variable region; VL, Light chain variable region; TAA, Tumor-associated antigen.

The bispecific T cell engager antibody (BiTE) with a small molecular size is a subtype of recombinant bispecific antibodies with two linked single-chain fragment variables (scFvs) derived from two distinct antibodies, one of which targets a pan T cell marker, In most cases CD3, and the other of which targets surface tumor-associated antigens (TAAs) (**Figure 1**) (29, 30). In cellular models, BiTEs has been found to exhibit significantly higher efficacy in tumor cell lysis compared to monoclonal IgG antibodies as well as other bispecific antibodies. The effectiveness of BiTE is reported to be Up to hundreds of times greater, even when the ratio of T cells to target tumor cells is limited (31). The production of BiTE has proven to be advantageous due to its ability to be generated in significant amounts by mammalian cell lines. This offers a relatively straightforward and efficient production process when compared to time-consuming and difficult methods like CAR T cells (21, 32).

One of the primary benefits of BiTE and TriTE molecules is their ability to provide “specificity” to polyclonally-activated populations of T cells, resulting in resistance to tumor immune evasion strategies, such as the downregulation of Major histocompatibility complex (MHC) molecules (33). MHC molecules play a crucial role in the presentation of TAAs. TAAs are processed by antigen-presenting cells such as dendritic cells and presents to the T-cell receptor (TCR) on T cells by MHC molecules. This interaction leads to the activation of T cells, resulting in eliminate of cancer cells. This process is commonly known as MHC restriction (34). The intrinsic resistance to immunotherapies, such as immune checkpoint inhibitors therapy, can be attributed to the impairment or loss of the ability to present antigens for MHC molecules (35, 36). This is a significant factor that impedes the effectiveness of these therapeutic approaches. In addition, it is important to note that the activation of T-cells and the subsequent immune response relies on the presence of costimulatory signals such as CD28 signaling (37, 38).

BiTE has the ability to bridge the gap between cytotoxic T cells and cancer cells, even in the absence of MHC restriction and costimulatory signals. By acting as a biological bridge, BiTE facilitates the activation and proliferation of T cells, regardless of MHC restriction, finally leading to the formation of the immunologic synapse (33, 39). Additionally, BiTE is not dependent on costimulatory signals for T-cell activation. Costimulatory signals, are typically required to fully activate T cells. However, BiTE can independently trigger T-cell activation, making it an adjustable mechanism in cancer immunotherapy (21, 40). **Table 1** shows some of the ongoing cancer clinical trials related to BiTEs and TriTEs.

**Table 1:** Clinical trials about bi- and tri-specific T cell engagers.

BiTE/TriTE Name	Targeted antigens	Condition	Phase of study	NCT	Status
**AMG330**	CD33 x CD3	AML	I	[NCT02520427]	Terminated
**AMG673**	Anti-CD33 with Fc domain	AML	I	[NCT03224819]	Terminated with results
**JNJ-63709178**	CD123 x CD3	AML	I	[NCT02715011]	Completed
**MCLA-117**	CLEC12AxCD3	AML	I	[NCT03038230]	Not applicable
**AMG420 (BI836909)**	BCMA	Multiple Myeloma	I	[NCT02514239]	Completed with results
**Solitomab (AMG110, MT110)**	Anti-EpCAM	Several solid tumors	I	[NCT00635596]	Completed
**AMG211 (MEDI-565)**	Anti-CEA	Gastrointestinal Adenocarcinomas	I	[NCT01284231]	Completed
**AMG757**	DLL3 x CD3	Small Cell Lung Cancer	I	[NCT03319940]	Recruiting with publication (41)
**AMG596**	EGFRvIII x CD3	Glioblastoma	I	[NCT03296696]	Completed with publication (42)
**BAY2010112**	PSMA x CD3	Prostate Cancer	I	[NCT01723475]	Completed
**BI 764532**	DLL3 x CD3	small-cell lung cancer and neuroendocrine carcinomas	I	[NCT04429087]	Recruiting with publication (43)
**Tebentafusp**	HLA-A*02:01 x CD3	Uveal Melanoma	II/III	[NCT03070392]	Active, not recruiting with publication (44, 45)
**SAR442257**	CD3xCD28xCD38	Multiple Myeloma, Non-Hodgkin lymphoma	I	[NCT04401020]	Recruiting

**Figure 2:**
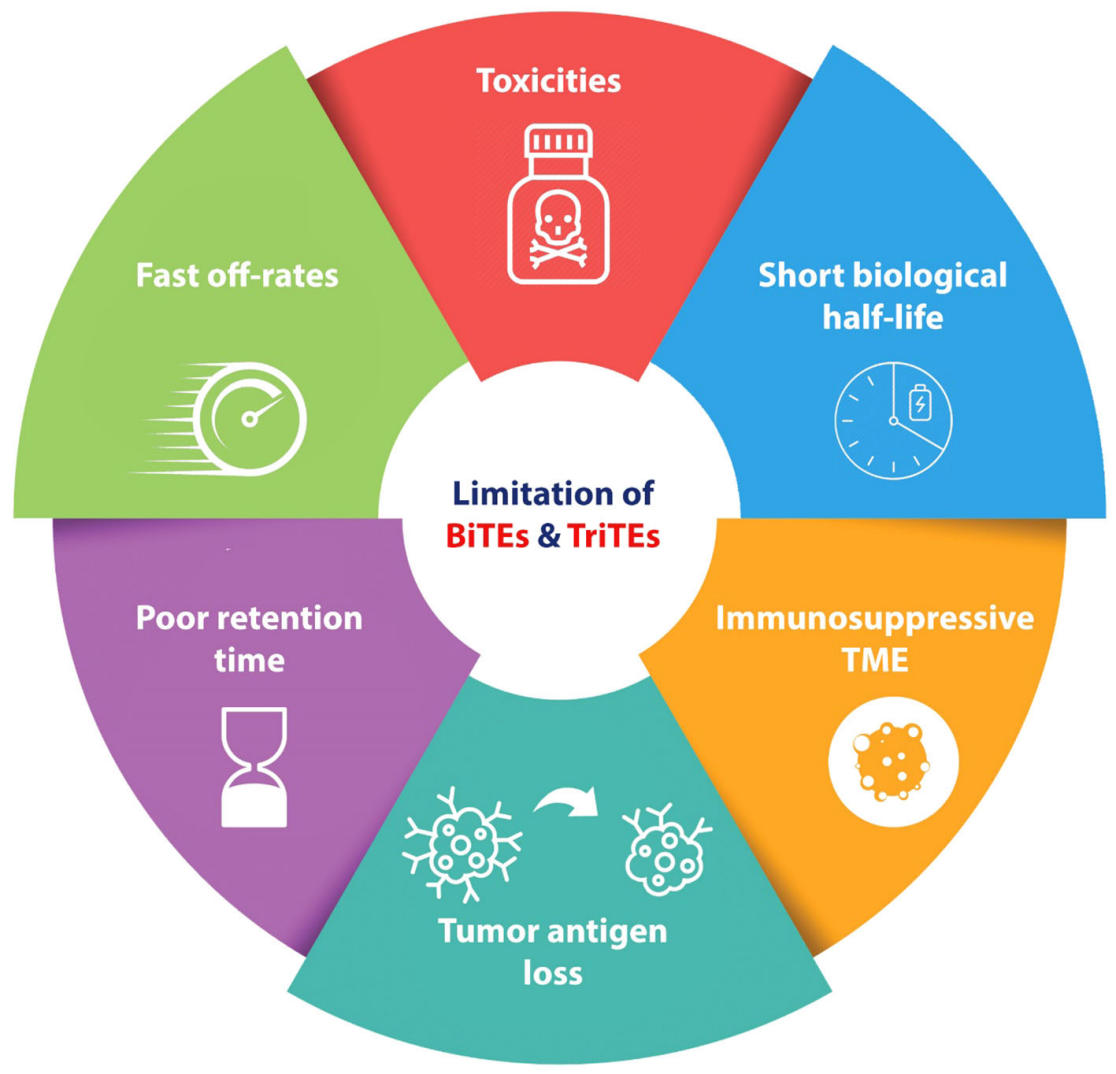
Schematic represents the disadvantages and challenges of BiTEs and TriTEs in pre-clinical and clinical cancer studies. BiTE, Bi-specific T Cell Engager; TriTE, Tri-specific T Cell Engager; TME, Tumor micro-environment.

The administration of BiTEs as a novel therapeutic approach for cancer treatment, similar to other procedures, is not without its disadvantages (**Figure 2**). One characteristic commonly observed in BiTEs/TriTEs is their short biologic half-lives and rapid blood clearance. This means that these molecules are rapidly metabolized. Additionally, they exhibit fast off-rates, which refers to their ability to dissociate from their target molecules quickly (46–48). Another important aspect to consider is the poor retention times of BiTEs/TriTEs in targeted tumor sites (48). While BiTEs have demonstrated efficiency in numerous cases of relapsed or refractory hematological malignancies, there is a subgroup of patients with hematological malignancies who do not exhibit a response to BiTEs therapy. To enhance the effectiveness of BiTEs, it is imperative to conduct more research regarding tumor escaping mechanisms.

The term of antigen loss refers to the absence of antigen expression and the inability of targeted antibodies or cells to bind to antigens. The occurrence of either or both of these conditions can result in a relapse characterized by the absence of CD19 expression in B-cell lymphoma as well as ALL (49–51). Targeted antigen loss has been identified as a significant factor in patients who have not responded to anti-CD19 CAR T cell treatment. This observation highlights the crucial role that antigen loss contributes in the development of resistance to T-cell based immunotherapies for tumors (50, 51). In a study conducted by Braig et al., the scientists studied patients with ALL who had received blinatumomab and subsequently experienced relapse characterized by the absence of CD19 expression (49). Thus, employing of multi-targeted approaches could prove advantageous for tackling antigen loss. This may involve the development of a singular pharmaceutical agent capable of simultaneously targeting several TAAs, or alternatively, the combination of diverse immunotherapeutic modalities, each targeting distinct TAAs.

The impaired function of the immune system, particularly T cells suppression, constitutes an important variable contributing to this phenomenon (52). For instance BiTE resistance may be related to PD-1/PD-L1 axis. Köhnke et al. in a case study on one B-precursor ALL patient who was resistant to treatment with blinatumomab (a CD19/CD3 bsAb antibody) demonstrated that, after blinatumomab treatment, PD-L1 expression was increased on the tumor cells, suggesting combination of BiTE therapy with programmed cell death protein 1/programmed death-ligand 1 (PD-1/PD-L1) inhibitors could be beneficial for managing tumor immune escaping mechanism (53). Further studies confirmed that the upregulation of immune checkpoints, particularly PD-L1, was observed following BiTE treatment in AML cells and among patients with diverse hematologic neoplasms. This suggests that the combination of immune checkpoint inhibitors with BiTE therapy represents an appropriate strategy to enhance BiTE-induced cytotoxicity (54, 55).

These issues are also observed in BiTEs Therapy for solid tumors as a result of the immunosuppressive tumor microenvironment such as dominance of immunosuppressive myeloid cells and increasing levels of Tregs (56–59). Also various types of solid tumor cells express the immune checkpoint proteins, which binds to the inhibitory receptors on T cells, consequently compromising the effectiveness of cellular immune responses (60). These obstacles go hand in hand, and consequently the efficacy of T cell-based immunotherapies, such as CAR T cell therapies and BiTE therapy, is compromised.

BiTE therapy, similar to other T-Cell based immunotherapies such as CAR-T cell therapy, has been associated with an elevated risk of toxicity as one of its adverse effects. Among the various adverse effects associated with BiTE therapy, two particularly concerning ones are cytokine release syndrome (CRS) and neurotoxicity (61–63). These adverse effects have been identified as having dose-limiting toxicities (DLTs), meaning that they can become severe enough to limit the dosage of the treatment (63). CRS is a pathological condition characterized by the upregulation of pro-inflammatory cytokines such as IL-6, and interferon-gamma (IFN-γ). The clinical and laboratory findings demonstrate a range of symptoms, including a mild cold-like symptomatology to a severe multi-organ failure, which has the potential to result in mortality (64).

The occurrence of neurological adverse effects can be related to the redistribution of activated T cells. The activation of T lymphocytes stimulated by BiTE results in their adherence to cerebral blood vessels and subsequent migration to the cerebrospinal fluid. The process of T cell sedimentation leads to the impairment of microcirculation and the development of local ischemia, finally giving rise to neurological symptoms (65).

In summary, despite the presence of both notable benefits and drawbacks associated with this innovative therapeutic approach, there exists considerable potential for further development of this category of molecules that orchestrate immune responses against malignancies. These molecules hold promise as cancer immunotherapeutic agents, particularly when applied in combination with OVs to overcome BiTEs/TriTEs monotherapy limitations.

## 3 Overview of the oncolytic viruses and cancer immunotherapy

**Figure 3:**
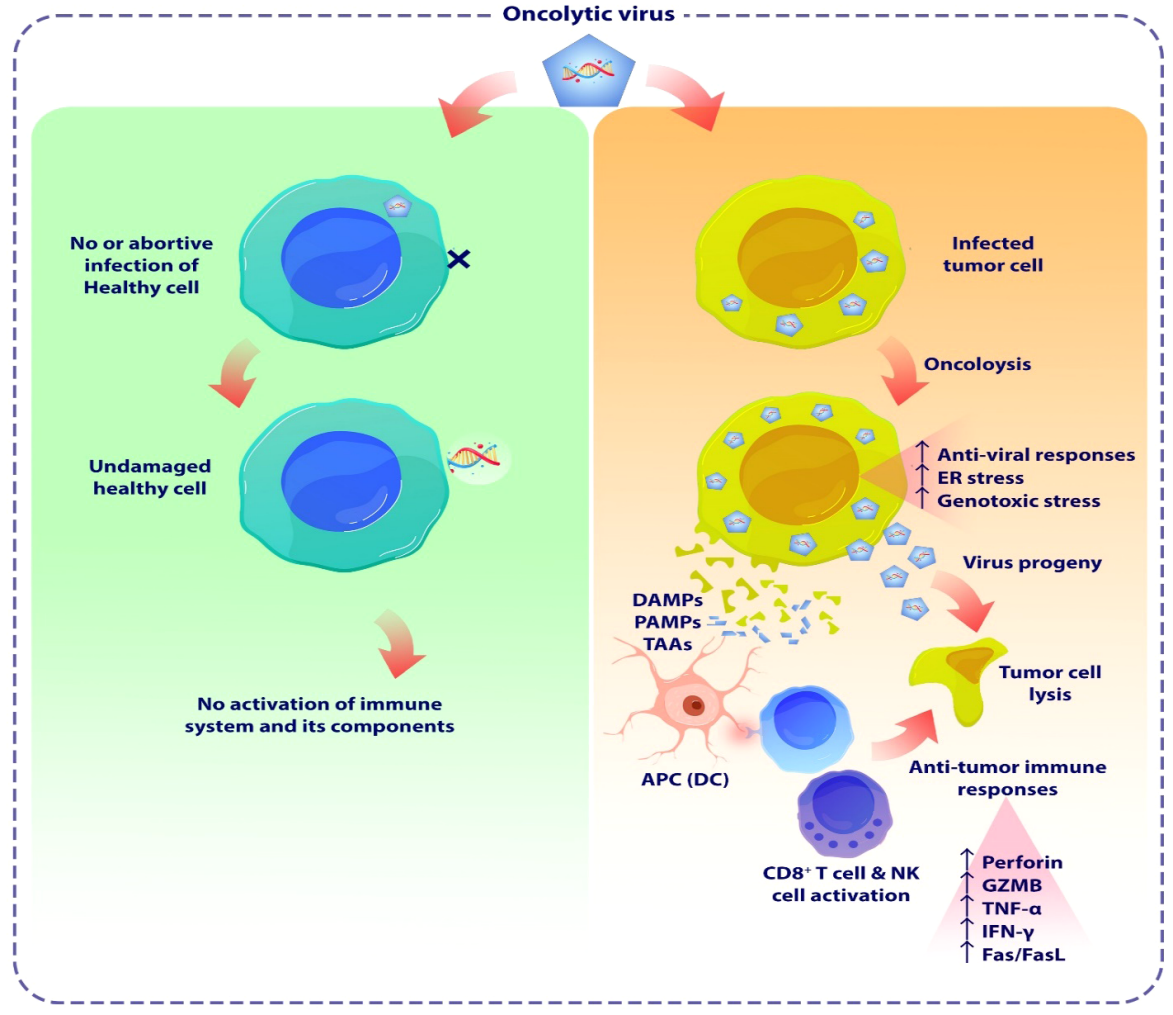
The schematic demonstrates the oncolytic viruses mediated tumor lysis. OVs are a novel class of cancer therapeutics that have the potential to eliminate tumor cells and enhance both the innate and adaptive immune responses specific to the tumor. OVs have been proven to trigger apoptosis, necroptosis, and autophagic cell death in malignant cells by many methods, encompassing both direct virus-mediated cytotoxicity and the stimulation of cytotoxic immune system pathways. OVs induce pro-inflammatory responses by enhancing the release of tumor antigens, leading to subsequent immune activation. OVs induce cellular damage and stimulate the release of DAMPs and PAMPs from lysed cancer cells. These substances activate PRRs in NK cells and macrophages, triggering them to secrete inflammatory cytokines such as IFN-γ, and TNF-α. Furthermore, the release of TAAs or TSAs from damaged tumor cells, subsequent presentation by APCs, stimulates adaptive immune responses, including the activation of antigen-specific CD4+ and CD8+ T cells. As a result, these T cells that preferentially target tumors can cause immunogenic cell death in cancer cells. OV, Oncolytic virus; DAMP, Damage-associated molecular patterns; PAMP, Pathogen-associated molecular patterns; TAAs, Tumor-associated antigens; TSAs, tumor-specific antigens; APC, Antigen presenting cell.

Since the 19th century, there have been several case reports of tumor regressions occurring simultaneously with natural viral infections. These patients primarily had hematological malignancies, such as leukemia or lymphoma, which are known to cause significant impairment of the immune system (66). During the 1950s and 1960s, our understanding of viruses greatly increased due to the substantial progress made in cell culture techniques. Virotherapy had attracted significant interest, with viruses such as hepatitis, West Nile, and Epstein-Barr virus commonly employed in cancer treatment at that time. Despite the varying and disputed outcomes (67–69), these reports yielded useful information. During the 1970s and 1980s, the use of viruses as a strategy for combating cancer was disregarded. However, after two decades, this type of treatment resurfaced and became known as “oncolytic viruses” (66). OVs, represent a pioneering category of cancer therapeutic approaches that facilitate the eradication of tumor cells while simultaneously enhancing the innate immune response and the tumor-specific adaptive immune response. OVs have been observed to induce cell death in cancer cells by multiple mechanisms, including direct virus-mediated cytotoxicity and the activation of cytotoxic immune system pathways (70, 71). The activation of the immune system occurs due to the release of cell debris and viral substances within the tumor’s surrounding environment. The selectivity of cancer cells in OV treatment is influenced by multiple parameters. One of these ways involves the entry of the virus into cells through specialized receptors that are specific to the virus. (**Figure 3**) (70, 72). It has been observed that Tumor cells have a propensity to express elevated levels of specific receptors such as CD46, ICAM-1, DAF, CD155, and integrins. These receptors play an essential role for OVs entry into the malignant cells within TME. For instance, in the case of glioblastoma multiform expressing the human poliovirus receptor CD155, administration of an oncolytic recombinant poliovirus (PVS-RIPO) through intrathecal delivery demonstrated a significant elevation in the median survival time among transgenic mice (73). Nevertheless, there are additional efforts to enhance the specificity of tumor targeting by redirecting OVs for entering cells via receptors that are specific to tumors. Furthermore, the rapid proliferation of tumor cells, characterized by elevated metabolic and replicative functions, may facilitate enhanced viral replication in comparison to normal, quiescent cells. Also, tumor-driver mutations notably enhance the virus replication in the cancer cells (74, 75). In addition, a large percentage of malignant cells demonstrate deficiencies in the signaling of antiviral type I interferon, hence promoting the replication of certain viruses (76).

Adenovirus (AdV), coxsackievirus, herpes simplex virus (HSV), Maraba virus, measles virus, Newcastle disease virus, parvovirus, reovirus, vaccinia virus (VACV), and vesicular stomatitis virus (VSV) are a few of the interesting OV platforms that are currently being tested in pre-clinical and clinical settings (77). OVs with DNA genome, demonstrate notable advantages due to their larger genome size, durable polymerase enzyme, genomic consistency, and strong proliferation capacity. RNA viruses, alternatively, exhibit outstanding compatibility for the purpose of selectively targeting tumor cells growths within the central nervous system, because of their smaller sizes and remarkable capacity to penetrate the blood-brain barrier (69).

The efficacy of OV immunotherapy depends on two pivotal factors: the capacity to selectively target neoplastic cells and the triggering of systemic immune system responses. OVs have the potential to exploit the unique susceptibilities of malignant cells, including their abnormal stress responses, signaling pathways, and homeostasis processes (78). These abnormalities, which have a possibility to impair the effective functioning of viral clearance mechanisms such as interferon (IFN), toll-like receptor (TLR), Janus kinase-signal transducer and activator of transcription (JAK-STAT), and protein kinase R (PKR) pathways, frequently make cancer cells vulnerable to OV invasion and replication, while protecting healthy cells from adverse effects (79).

OVs frequently trigger immunogenic cell death in cancer cells, and they have the potential to directly engage with immune cells, thereby initiating an anticancer immune response (80, 81). These viruses interact with the immune system while they begin replication within solid tumors (80, 82). OVs cause cellular damage and promote the release of pathogen- and damage-associated molecular patterns (PAMPs and DAMPs) from lysed malignant cells (20). These molecules stimulate innate immune responses in NK cells and macrophages through pattern recognition receptors (PRRs), leading to the secretion of inflammatory cytokines like IFN-α, IFN-γ, TNF-α, IL-6, and IL-12 from these cells (83). Moreover, the release of tumor-associated antigens (TAAs) or tumor-specific antigens (TSAs) from damaged tumor cells and their subsequent presentation by antigen-presenting cells (APCs) stimulate adaptive immune responses, which involve the activation of antigen-specific CD4+ and CD8+ T cells (84, 85). Subsequently, these T cells that specifically target tumors can trigger immunogenic cell death in tumor cells, as confirmed in a preclinical investigation (86, 87).

The emphasis of early virotherapy studies on the inherent mechanism of oncolysis has led to the discovery of tumor-selective virus-mediated apoptosis, which presents an attractive alternative method of cancer therapy in the form of OVs. The reproduction capacity of OVs in healthy cells is limited, whereas the virus can selectively replicate in cancerous cells, leading to their lysis (88, 89). In addition to induction of apoptosis, cellular autophagy mechanisms is influenced by OVs in infected tumor cells. Upon viral infection, OVs interfere with the autophagy machinery in various tumor cells, impacting the self-degradation process (90). These mechanism suggest that inducing the autophagic process alongside virotherapy can improve anti-tumor efficacy in different types of cancer, including lymphoma, myeloma, leukemia, and brain cancers (87, 91, 92).

Nevertheless, it is important to note that although OVs can induce immune responses against cancer, an excessive antiviral response could impair the replication of OVs and may significantly reduce the efficacy of this therapeutic approach (73). Therefore, it is imperative to establish an equilibrium between the immune system responses and the oncolytic activities within the tumor microenvironment.

Currently, there are only two approved OVs for clinical use worldwide. Oncorine (H101) received approval from Chinese authorities in 2005 for treating nasopharyngeal carcinoma in combination with chemotherapy. In 2015, the FDA approved T-VEC (Talimogene laherparepvec) for the treatment of advanced melanoma patients in the United States (93, 94). Several OVs, including HF10 (Canerpaturev), CVA21 (CAVATAK), and Pexa-Vec (a vaccinia virus), are currently undergoing phase II/III clinical trials either as monotherapy or in combination with immune checkpoint inhibitors for several malignancies (70, 95–97). Moreover, there is provisional regulatory approval in Japan for the HSV-based OV called Delytact (Teserpaturev/G47Δ), which is a genetically modified third-generation herpes simplex virus type 1 (HSV-1) with triple mutations, primarily for treating malignant gliomas (98, 99). These advancements represent notable progress in the field of OV therapy.

The utilization of OVs in cancer therapy poses several challenges, including the issues of Limited Virus Penetration, patient selection, passive targeting, immune responses and hypoxia (100–102). In order to overcome these challenges that OV monotherapy is facing, today the attention of scientists in this field has been drawn to the genetic engineering of these viruses in order to express cytokines, chemokines, and also recombinant antibodies. These changes can significantly increase the efficiency of this novel therapeutic approach.

## 4 The applications of oncolytic viruses for the delivery of immunotherapies

Traditionally, the primary treatment methods employed in cancer management have mainly limited and consisted of combinations of chemo-radiotherapy, surgical intervention, and targeted therapies. Despite the continuous progress achieved in developing various therapeutic strategies, reducing the risk of adverse effects resulting from these procedures still poses considerable issues (69). It has been determined that immunotherapy, particularly via the application of immune checkpoint inhibitors (ICIs), CAR-T cells, monoclonal antibodies (mAb), and bispecific molecules, is not exempt from this concept (**Table 2**). Numerous patients experience significant adverse effects, such as auto-inflammatory disorders and autoimmunity, which develop from the non-specific stimulation of the immune system and unintended impacts on non-targeted tissues. Therefore, there exists considerable potential for the application of OVs to enhance the safety and specificity of addressed therapeutic interventions, primarily by precisely and exclusively conducting these antibodies toward the tumor site (69, 105, 106). **Table 2** presents a brief summary of mentioned immunotherapies currently used for cancer treatment, highlighting the challenges associated with monotherapy as well as the numerous advantages associated with the application of modified OVs. The table also includes the cancer clinical trials on these OVs.

**Table 2:** Summary of immunotherapies in cancer treatment: monotherapy challenges, modified oncolytic viruses, and related clinical trials.

Type of immunotherapy	Description	Disadvantages of monotherapy	Advantages of modified OVs	Clinical trials
**Therapeutic antibodies**	Therapeutic antibodies, such as ICIs, function by blocking homeostatic signals, such as CTLA-4 and PD-1, with the aim of triggering immune responses against tumor cells (103).	- Limited efficacy in some patients - ICIs are less effective in treating ‘‘cold’’ tumors - Pulmonary and Gastrointestinal toxicities - Neurologic and ocular complications - Rheumatologic complications - Dermatological toxicities	- Enhanced therapeutic response - OVs modulate TME to make “cold” tumors susceptible to immune checkpoint inhibitors. - Improved tumor lymphocyte infiltration - Improved tumor penetration - Reduced side effects through local delivery	- NCT05788926 (Recruiting/Phase I): TG6050 (CTLA-4 antibody) is an oncolytic vaccinia viruses/Non-small cell lung cancer - NCT04336241 (Recruiting/Phase I), NCT05733611 (Active, Not recruiting, Phase II): RP2 (CTLA-4 antibody) is a genetically modified HSV-1/Metastatic Colorectal Cancer - NCT05081492 (Active, Not recruiting, Phase I): CF33 (hNIS/Anti-PD-L1 antibody) is a genetically modified Orthopoxvirus/Metastatic Triple Negative Breast Cancer - NCT05733611 (Active, Not Recruiting, Phase II), NCT05733598 (Not yet recruiting, Phase II), NCT05743270 (Withdrawn, Phase II), NCT04735978 (Active, Not yet recruiting, Phase I): RP3 (CTLA-4 antibody) is a genetically modified HSV-1/Metastatic Colorectal Cancer, Squamous Cell Carcinoma of Head and Neck, and Hepatocellular Carcinoma - NCT03852511(Completed, Phase I): NG-350A (Anti-CD40 antibody) is a genetically modified Adenovirus/Advanced Epithelial Tumors
**CAR T cells**	CAR T cell therapy is a form of cancer immunotherapy wherein T cells, undergo genetic engineering to enhance their ability to identify and eliminate tumor cells with improved efficacy (104).	- Limited success in solid tumors - Antigen escape - Limited tumor infiltration - Tumor heterogeneity - CAR-T cell toxicity (e.g. CRS and ICANS) - T cell exhaustion - On-target off-tumor effects - Immunosuppressive TME limited the efficiency of CAR T cell	- Increased CAR T cell infiltration - Reducing tumor immune escaping - Increase the T cells activity to suppress tumors and increase the lifespan - Improved efficacy by combination therapy with cytokine-armed OVs (e.g., IL-2, IFNs) - OV-mediated delivery of tumor-selective surface antigens enhances the antitumor efficacy of CAR T-cells - OVs modulate the TME via enhancing the expression of immune checkpoint costimulatory receptors and ligands. (e.g., OX40, OX40L, 4-1BB, 4-1BBL)	- NCT03740256 (Recruiting): CAdVEC/A First in Human Phase I Trial of Binary Oncolytic Adenovirus in Combination With HER2-Specific Autologous CAR T Cells in Patients With Advanced HER2 Positive Solid Tumors
**Bi- and Tri-specific molecules**	BiTE is a recombinant bispecific antibody containing two linked scFvs derived from distinct antibodies. One scFv targets a T cell-surface molecule, while the other targets cancer cell antigens. TriTEs are capable of identifying three distinct targeted antigens (21, 22).	- Short biological lifespan - Poor tumor retention - Antigen escape - Limited memory immune response - toxicity such as CRS	- Activated T/NK cells for tumor lysis - Enhanced tumor specific targeting - Enhanced tumor cytotoxicity both *in vitro* and *in vivo* - Significant reduction in tumor growth *in vivo* - Prolonged remission of tumors without recurrence in animal models	- NCT05938296 (Recruiting/Phase I): BS006 (PD-L1/CD3-BsAb) is a genetically modified HSV-2/Metastatic Solid Tumors

ICI, Immune checkpoint inhibitor; OV, Oncolytic virus; HSV, Herpes simplex virus; TME, Tumor micro-environment; CRS, Cytokine release syndrome; ICANS, Immune effector cell-associated neurotoxicity syndrome; IFN, Interferon.

## 5 The incorporation of bi- and tri-specific t cell engagers into oncolytic virotherapy

As described in previous sections, T cell engagers including BiTEs and TriTEs has attracted considerable interest among physicians and scientists. However, their short serum half-life mandates continuous infusion, and systemic administration can lead to severe and fatal side effects. Also, efficacy of this therapeutic approach against solid tumors is constrained by tumor barriers and immune-suppressive microenvironments (9, 107–109). One of the approaches that received considerable interest in the field of cancer immunotherapy to address these limitations is oncolytic virotherapy.

**Figure 4:**
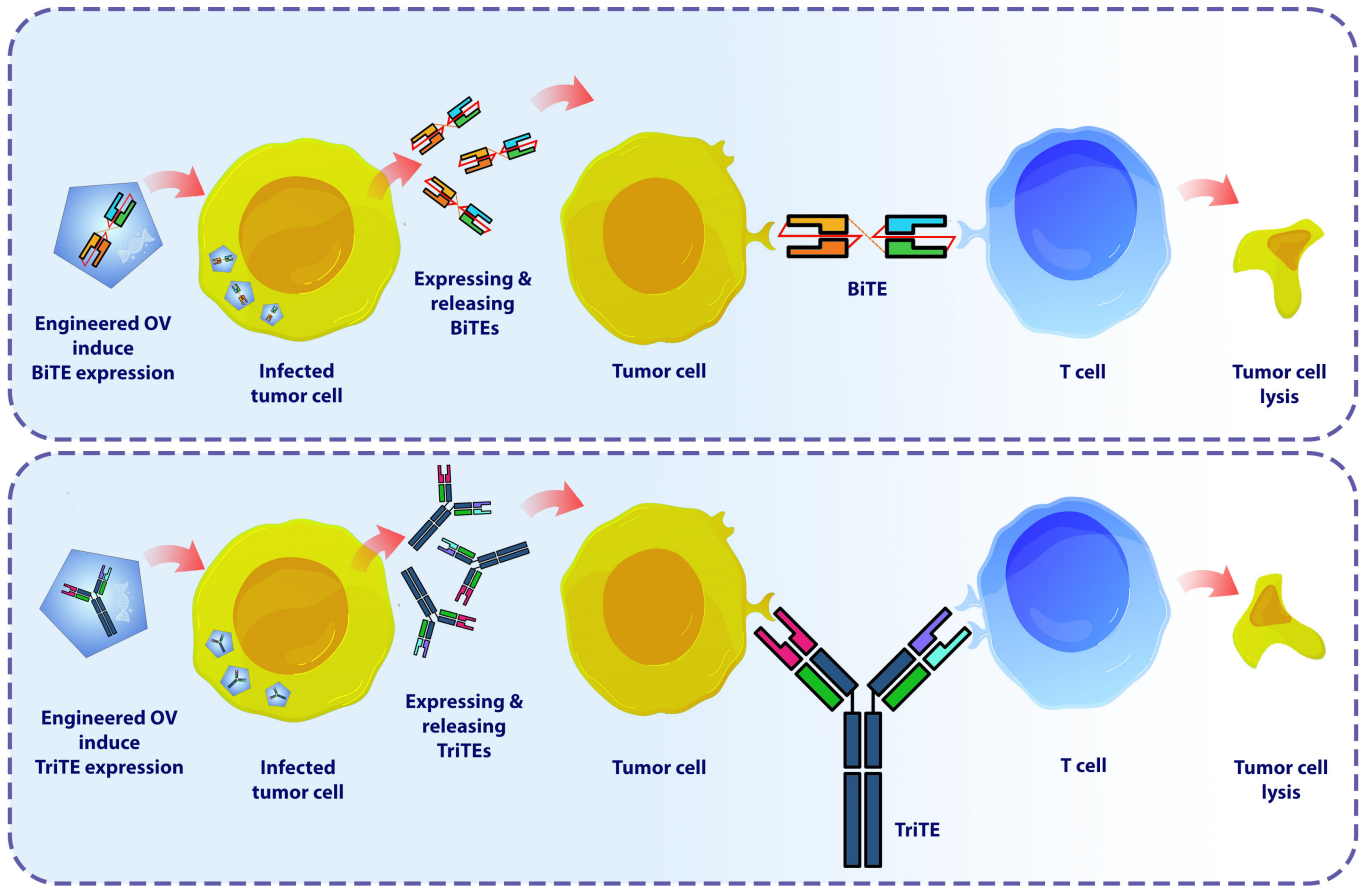
The schematic demonstrates how OV-BiTEs/TriTES work against cancer cells. The production stages (Not shown) for engineered OVs employed as vectors for expressing BiTEs and TriTEs in Studies in this field adhere to a standardized approach. These OVs contain transgenic cassettes that encode bi- or tri-specific T cell engagers. Oncolytic-modified viruses possess the ability to selectively target and damage cancer cells. Infected tumor cells secrete BiTEs and TriTEs antibodies, which serve as attractants for T lymphocytes, hence facilitating their recruitment to the tumor microenvironment. In the context of this therapeutic approach, alongside the viral-mediated lysis of tumor cells, the trigger of tumor cells killing is also attributed to the presence of specific T lymphocytes. BiTE, Bi-specific T cell engager; TriTE, Tri-specific T cell engager.

The combination of BiTEs/TriTEs with OVs holds the potential for mutual advantages. The infection caused by OV triggers a localized inflammatory response and attracts T cells to the tumor site. These T cells can be guided towards tumor cells by the administration of BiTEs (**Figure 4**) (71, 73, 110–112). Furthermore, the use of OVs for encoding BiTEs/TriTEs is an opportunity to address the limitations associated with BiTEs/TriTEs therapy. This delivery method has the potential to enhance the concentration of this therapeutic molecules specifically at the site of the tumor and facilitate its penetration into solid tumors, all the while minimizing its distribution within the body and systemic exposure (113–115). Consequently, this approach enhances the therapeutic efficacy by improving the range of doses that could be safely administered.

Typically, the production stages for engineered OVs employed as vectors for expressing BiTEs and TriTEs in Studies in this field adhere to a standardized approach. These OVs contain transgenic cassettes that encodes bi- or tri-specific T cell engagers. Typically, the BiTEs/TriTEs sequences are composed of scFvs that are developed to specifically bind to CD3, together with either a TAA or antigens expressed on cancer-associated fibroblasts or tumor-associated macrophages (116–118). Regulatory domains such as promoters and leader sequences encoding secretory signaling peptides derived from immunoglobulins are commonly found upstream of transgenes (113). Subsequently, the viral vectors and transgenic products are subjected to characterization. The assessment of viral replication kinetics and the potential for direct tumor cell killing involves quantifying progeny and employing several cell viability assays, including metabolic, impedance, or flow cytometry-based evaluations. The confirmation of the expression and secretion of BiTEs/TriTEs is achieved through SDS-PAGE and immunoblotting procedures on the cell-free supernatant obtained from virus-infected cells. In order to determine the binding specificity of BiTEs/TriTEs towards their target antigens and cells expressing the antigens, researchers used ELISA and/or flow cytometry tests. Furthermore, the functionality of BiTEs/TriTEs is explored through *in vitro* co-culture experiments involving target cells and immune effector cells (117, 119–123). In **Table 3**, engineered OVs expressing BiTEs/TriTEs and targeted antigens along with the results and observations of each study are summarized.

**Table 3:** BiTE- and TriTE-armed OVs for cancer immunotherapy in pre-clinical studies.

OV type	OV-BiTEs name	Targeted Antigen (s)	Results and observations	References
**AdV**	OAd-MUC16-BiTE	CD3 x MUC16	- MUC16-BiTE mediated T cell activation and target cancer cells specially. - OAd-MUC16-BiTE–mediated enhanced T-cell–mediated tumor cell killing and bystander effect. - OAd-MUC16-BiTE enhanced infiltration of CTLs and reversed the immunosuppressive TME. - Promoted T cell trafficking to the tumor by increasing pro-inflammatory factors and decreasing anti-inflammatory factors. - Local administration lowered toxicity and systemic exposure.	(119)
	ICOVIR-15K-cBiTE	CD3 x EGFR	- ICOVIR-15K-cBiTE mediated robust T cell activation, proliferation, and bystander cell-mediated cytotoxicity. - ICOVIR-15K-cBiTE Increased TIL durability and accumulation *in vivo*. - ICOVIR-15K-cBiTE Improved antitumor activity when coupled with PBMCs or T cells.	(116)
	ICOVIR-15K-cBiTE	CD3 x EGFR	- ICOVIR-15K-cBiTE mediated robust T cell activation, proliferation, and cytotoxicity. - ICOVIR-15K-cBiTE increased antitumor effectiveness when combined with PBMCs or T cells in xenograft models. Mesenchymal stem cells are being employed as carriers to enhance delivery.	(120)
	EnAdV	CD3 x EpCAM	- EpCAM-BiTE mediated activation of CD4+ and CD8+ T‐cell populations. - EnAdV increased TIL infiltration and mediated long-lasting antitumor immunity. EnAdV- EpCAM BiTE overcome immunosuppressive environment and enhanced activation of endogenous T cells.	(121)
	EnAd-FAP-BiTE	CD3 x FAP	- EnAd-FAP-BiTE mediated T cell activation, their subsequent proliferation, and the induction of cytotoxicity in cancer cells. - EnAd-FAP-BiTE induces repolarization of resident TAMs in ascites samples. - EnAd-FAP-BiTE increased infiltration of T cells.	(124)
	ICO15K-FBiTE	CD3 x FAP	- Supernatants from ICO15K-FBiTE-infected cells triggers the activation and proliferation of T lymphocytes. - ICO15K-FBiTE promoted tumor T-cell retention and accumulation *in vivo*. - ICO15K-FBiTE is more effective in combatting tumors than the parental virus.	(125)
	–	- CD3ϵ x (CD206 or FRβ) - CD3ϵ x CD3ϵ x CD206 - CD3ϵ x CD28 x CD206	- Selective elimination of M2-polarized autologous macrophages as opposed to M1-polarized autologous macrophages. - A TriTE, possessing bivalent CD3ϵ binding, showed enhanced efficacy while maintaining its selectivity towards target cells. In contrast, a TriTE containing CD28 has induced non-specific activation of T cells. - Enhanced activation of endogenous T cells and IFN-γ production upon exposure to both free and EnAd-encoded T cell engagers.	(122)
	ICO15K-cBiTE	CD3 x EGFR	- ICO15K-cBiTE –mediated oncolysis enhances activation and proliferation of CAR-T cells. - CAR-T cells in combination with ICO15K-cBiTE enhances antitumor efficacy and T cell activation *in vivo*.	(126)
	CAdTrio	CD3ϵ x CD44v6	- CAdTrio Enables T Cells to Kill Tumor Cells *In Vitro*. - CAdTrio Increases the Anti-tumor Activity of HER2.CAR-T Cells.	(127)
**VV**	OVV-CD19 BiTE	CD3 x CD19	- Supernatants from OVV-CD19 BiTE -infected cells induces the activation and proliferation of T lymphocytes. - Long-term tumor remissions without recurrence noted. - OVV-CD19 BiTE triggers T cells proliferation and recruited this lymphocytes to the tumor sites. - OVV-CD19 BiTE has higher anticancer activity than parental virus and blinatumomab.	(128)
	EphA2-TEA-VV	CD3 x EphA2	- EphA2-TEA-VV infected tumor cells induced T cell activation. - EphA2-TEA-VV redirects T lymphocytes to EphA2-positive cancer cells. - EphA2-TEA-VV induces bystander killing of non-infected tumor cells and enhanced antitumor activity *in vivo*.	(117)
	mFAP-TEA-VV	CD3 x mFAP	- mFAP-TEA-VV replicated within tumor cells and induced oncolysis similarly to the unmodified VV. - mFAP-TEA-VV demonstrates significant anticancer efficacy in an immunocompetent B16 melanoma model.	(118)
	VV-EpCAM BiTE	CD3 x EpCAM	- The secretion of EpCAM BiTE has been shown to effectively promote activation of T cells. - The VV-EpCAM BiTE demonstrates enhanced antitumor activity in EpCAM-expressing breast cancer. - Both VV-EpCAM BiTE and VV-Ctrl exhibit similar anticancer properties in the EpCAM-negative carcinoma model.	(129)
**Measles viruses**	MV-BiTE	CD3 x (CEA or CD20)	- Therapeutic efficacy of MV-BiTE in combatting malignancies in immunocompetent mice. - An association between the efficacy of anti-tumor agents and the enhanced presence of TIL.	(123)
**HSV**	oHSV-1 PD-L1 BiTE	CD3 x PD-L1	- PD-L1 BiTE does not increases killing of activated T cells. - oHSV-1 PD-L1 BiTE overcome immune-suppressive ascites fluids environment and have toxic effect for tumor cells. - oHSV-1 PD-L1 BiTE polarized M2-like macrophages. - oHSV-1 PD-L1 BiTE activate endogenous T cells.	(130)
	OV-mOX40L	CD3 x OX40L	- OV-mOX40L inhibited tumor growth *in vivo* - Local treatment of OV-mOX40L stimulated intratumoral immune cells. - OV-mOX40L activated CD4+ T cells and CD8+ cytotoxic T cells and reduced Treg proportion leading to switching the TME to a more pro-inflammatory state.	(131)
	- oHSV2-BiTEs-PD-L1 - oHSV2-mBiTEs-CD19	- CD3 x PD-L1 - CD3 x CD19	- oHSV2-BiTEs-PD-L1 mediated T cell activation boosting T cell cytotoxicity. - oHSV2-BiTEs-PD-L1 strengthens antitumor activity.	(132)

AdV, Adenovirus; VV, Vacina virus; HSV, Herpes simplex virus; OV, Oncolytic virus; BiTE, Bi-specific T cell engager; MUC-16, Mucin 16; EGFR, Epidermal growth factor receptor; TME, Tumor micro environment; TIL, Tumor infiltrated lymphocyte; PBMC, peripheral blood mononuclear cells; EpCAM, Epithelial cellular adhesion molecule; TAM, Tumor associated macrophage; FAP, Fibroblast activation protein; FRβ, Folate receptor-β; EphA2, Ephrin type-A receptor 2; CEA, carcinoembryonic antigen; PD-L1, Programmed death-ligand 1; OX40L, OX40 ligand.

This type of combination therapy is a recent innovation that originated within the past ten years. In 2014, Yu et al. conducted a pioneering study wherein they employed an oncolytic Vaccinia virus (VV) that harbored a T cell engager specifically designed to target EphA2 (EphA2-TEA-VV) (117). This BiTE construct had previously demonstrated a capability to selectively target and effectively suppress tumor growth (133). The study was carried out on a lung cancer mouse models, which expressing the tumor antigen EphA2. Administration of this therapeutic construct resulted in the significant inhibition of tumors growth, whereby such outcomes were simultaneously associated to the upregulation of effector cytokines. Tumor cells that were infected with EphA2-TEA-VV induced the activation of T cells, as indicated by the release of IFN-γ and IL-2. The results of *in vivo* experiments demonstrated that the application of EphA2-TEA-VV, in combination with the adoptive transfer of human T cells, resulted in significantly enhanced antitumor efficacy compared to the control group receiving VV plus T cells. Hence, the application of BiTE-armed OVs is a highly encouraging strategy to enhance the efficacy of oncolytic immunotherapy (117). Nevertheless, the methodology still requires evaluation in models that closely resemble clinical conditions, wherein the presence of intratumoral T cell infiltration and immunosuppressive TME are commonly evaluated (134).

In another research published three years after the initial study ICOVIR-15K-cBiTE, an oncolytic adenovirus (AdV) expressing a BiTE targeting the epidermal growth factor receptor (EGFR), was examined (116). Fajardo et al. used a scFv made from and FDA approved monoclonal antibody cetuximab, which is effective against metastatic colorectal cancer (116, 135). ICOVIR-15K-cBiTE has demonstrated significant oncolytic properties, leading to the activation and proliferation of T cells. Furthermore, this approach has also been found to facilitate bystander cell-mediated cytotoxicity, thereby enhancing its therapeutic potential. *In vivo* studies demonstrated a significant increase in the tumor-infiltrated lymphocytes (TILs) and retardation in tumor growth in tumor xenograft models treated with ICOVIR-15K-cBiTE, in comparison to mice given the primary virus and the control group. The immunohistochemical assessments demonstrated comparable levels of viral proteins in all groups that were treated with the virus, irrespective of the administration of peripheral blood mononuclear cells (PBMCs). This suggests that the virus remains present at the tumor site despite the existence of effector T cells. Also, the cBiTE-mediated cancer cell death does not have any negative impact on the virus’s capacity to persist in the tumor, as observed in the animal models (116). Generally, the results of this study reveals that BiTE-armed oncolytic adenoviruses possess distinct characteristics that can stimulate targeted and redirected immune responses against tumors. This approach demonstrates a capacity to address significant constraints in oncolytic virotherapy. Another research has provided more evidence that an EGFR-targeted BiTE armed OV can be effectively delivered into the TME by utilizing mesenchymal stem cells (MSCs) as carriers, resulting in enhanced therapeutic effectiveness and systemic availability of ICOVIR-15K-cBiTE. The findings of the study demonstrate the successful production of cBiTE from OAd-infected MSCs, resulting in enhanced cytotoxicity both *in vitro* and *in vivo*. These results confirm the effectiveness of the synergistic effect of OAd, cBiTE, and MSCs in controlling tumor growth. The comparison of *in vivo* antitumor efficacy between the cBiTE-expressing and non-expressing OAdv in combination with MSCs is of particular significance to this study. While the group treated with MSCs/ICOVIR15-cBiTE showed a significant reduction in tumor growth compared to other treatment groups, MSCs/ICOVIR15 also demonstrated improved tumor growth control compared to the ICOVIR15 groups (ICOVIR15, ICOVIR15-cBiTE) and untreated mice. These findings indicate that using ICOVIR15-cBiTE in combination with MSCs may present a promising strategy for cancer treatment that warrants further investigation in clinical trials (120).

In the study published by Wang et al., researchers made modifications to the parental Oncolytic Adenovirus by expressing a MUC16-targeting BiTE antibody. This modified variant, known as OAd-MUC16-BiTE, demonstrated that it maintained its oncolytic properties and ability to replicate *in vitro*. The BiTE molecule, released by tumor cells, accumulates within the TME. It has the ability to bind MUC16 located on targeted cells, subsequently forming connections with CD3 receptors present on T cells. This interaction triggers a series of events, including the activation, proliferation, and damaging effects of T cells against tumor cells that express MUC16. In ex vivo tumor cultures that were obtained from patients with ovarian cancer, OAd-MUC16-BiTE, successfully overcame the immunosuppressive TME. As a result, it displayed enhanced cytotoxicity compared to the wild type virus. Furthermore, in the context of cell-derived xenograft and patient-derived xenograft models, OAd-MUC16-BiTE demonstrated heightened antitumor efficacy and a notable augmentation in CTLs, as compared to the primary virus. In summary, the combined use of OVs and MUC16-BiTE provides a synergistic effect that overcomes its drawbacks. This approach offers a new and innovative therapeutic option for ovarian cancer. In addition, it can be utilized in combination with diverse cancer treatments, including immune checkpoint inhibitors, chemotherapy, and VEGF inhibitors; nevertheless, investigations evaluating clinical efficacy are necessary (119).

The OV-BiTE strategy has yet to be demonstrated to be effective in more realistic immunological context models. Freedman et al. applied modifications to the oncolytic group B adenovirus EnAdenotucirev (EnAdV) in order to facilitate its capacity to express an additional BiTE. The BiTE construct has been engineered to exhibit dual binding affinity for EpCAM+ tumor cells and CD3+ T cells, leading to the formation of clusters and subsequent activation of CD4+ and CD8+ T cells alongside with cancer cells. In this study, the regulation of BiTE transcription is mediated by the primary late promoter of the virus, hence confining its expression to cancer cells that are capable of supporting virus replication. This methodology has the potential to enhance the cytotoxic effects of EnAd. This report showcases the application of this approach in primary pleural effusions and peritoneal malignant ascites, where the infection of cancer cells with BiTE-expressing EnAd triggers the activation of endogenous T cells. Consequently, these activated T cells are able to effectively eliminate endogenous tumor cells, even in the presence of an immunosuppressive TME. Overall, EnAd has the ability to encode bispecific T-cell engagers without compromising its oncolytic pathogenicity, thus showcasing its transgenic packaging capability. The transgene will not have any impact on the physicochemical characteristics of the viral particles. Therefore, the modified viruses are expected to exhibit identical clinical pharmacokinetics as their parental agent. Additionally, they will preferentially express the encoded BiTE specifically in tumors located throughout the body. The clinical studies of this novel and promising systemically targeted cancer immunotherapy should be given priority (121).

In another study Min Wei et al. engineered an oncolytic vaccinia virus expressing EpCAM Bispecific T-Cell Engager. The VV-EpCAM BiTE has demonstrated notable efficacy in the infection, replication, and lysis of tumor cells. The EpCAM BiTE molecule effectively formed a binding interaction between EpCAM-positive tumor cells and CD3ϵ receptors on T cells, subsequently initiating the activation of naive T cells and the subsequent release of various pro-inflammatory factors, including IFN-γ, IL2, IL6, and IL10. The administration of intratumoral injection of VV-EpCAM BiTE demonstrated a significant enhancement in the efficacy of tumor suppression within EpCAM-positive tumor models, when compared to the administration of wild type of vaccinia virus. Furthermore, there was a significant enhancement in the infiltration of immune cells within the TME in the group that received VV-EpCAM BiTE (129). The findings of this study provide evidence that BiTE-armored oncolytic VVs possess distinct characteristics that can stimulate targeted and redirected immune responses against tumors. The implementation of this particular strategy demonstrates the capacity to effectively overcome significant constraints in the application of oncolytic virotherapy and BiTE therapies within solid tumors. Consequently, it serves as a catalyst for the continued assessment and advancement of these therapeutic approaches.

In a separate investigation, cancer cells were treated by an engineered oncolytic measles virus expressing MV-BiTEs designed to target the tumor antigens CEA and CD20. As a result, the cancer cells were shown to release BiTE antibodies that exhibited functional properties. Significantly, the researchers demonstrated the therapeutic efficacy of MV-BiTE in combatting well-established malignancies in mice with completely functional immune systems. The present model demonstrate an association between the efficacy of anti-tumor agents and the enhanced presence of TIL, as well as the production of durable protective antitumor immune responses. Moreover, the therapeutic efficacy of MV-BiTE in xenograft spheroid models of patient-derived primary colorectal cancer was demonstrated when delivered in combination with human PBMCs. This study reveals the prolonged remission of tumors without recurrence and the development of immune protection following MV-BiTE therapy. This study demonstrates the feasibility of employing an oncolytic vector to express targeted BiTE, showing effectiveness against solid tumors (123).

Instead of directly focusing on cancer cells, BiTEs have the potential to be engineered in a manner that enables T cells to be directed towards pro-tumorigenic factors within the TME. Fibroblast activation protein-α (FAP) demonstrate an elevated expression level in CAFs, which serve as the predominant component of the tumor stroma. As a result, numerous researchers have employed FAP as a focal point for BiTE engineering (118, 136). CAFs exhibit diverse immune-modulating and pro-tumorigenic features, which encompass the secretion of transforming growth factor beta (TGF-β). These CAFs can be effectively addressed by targeting FAP which is known to be expressed on fibroblast cells that are involved in the natural healing process of wounds and tissue remodeling. Nevertheless, the delivery of FAP-BiTEs specifically to tumors by developing engineering OVs may offer an opportunity for minimizing any potential toxicities associated with non-selective targeting. A Vaccinia-based vector, known as mFAP-TEA-VV, was produced in a manner similar to the methodology employed for the development of EphA2-TEA-VV as discussed in the previous study (117, 118). In an immunocompetent melanoma model, mFAP-TEA-VV revealed significant anticancer activity when compared to control VVs and exhibited robust expansion in tumor sites. It is important to note that the increased viral spread caused by mFAP-TEA-VV had a favorable association with the elimination of tumor stroma. To summarize, this study offers preclinical evidence supporting the therapeutic advantages of TEA−VVs that target FAP on CAF. This study demonstrates that mFAP-TEA-VVs significantly increased the replication of viruses within tumors and exhibited potent anticancer effects in a mouse model of melanoma with an optimally functioning immune system (118).

Following this similar concept, Freedman et al. developed an EnAd-derived OV vector encoding a FAP-specific BiTE, capable of concomitantly targeting malignant and immunosuppressive stromal cells. This T-cell Engager shows a high affinity for FAP-expressed CAFs and CD3ϵ expressed on T cells. This interaction triggers a cascade of events, including the induction of fibroblast cell death and the efficient activation of T cells. In summary, EnAd-FAP-BiTE, in contrast to control vectors, resulted in enhanced activation of T cells and cytotoxicity. This led to the decrease of FAP-positive cells and subsequent reductions in TGF-β levels in ascites cultures. The mentioned effects were not detected in patient samples without cancer cells, thereby suggesting enhanced safety attributable to the vector’s precise tumor-directed ability. EnAd-SA-FAP-BiTE demonstrated a remarkable ability to enhance T cell-associated chemokines and effector molecules, while concurrently increasing the expression of genes implicated in dendritic cell maturation and antigen presentation across multiple biopsies. This observation implies the possibility of antigen dissemination and subsequent activation of diverse endogenous T cells, thereby facilitating the development of persistent anti-tumor immune responses. Furthermore, the administration of EnAd-SA-FAP-BiTE has been observed to induce the reprogramming of TAMs by altering their phenotypic expression from pro-tumorigenic M2 macrophages to a more pro-inflammatory M1 phenotype. Furthermore, the administration of this FAP-BiTE-encoding OV to freshly collected clinical biopsies, such as malignant peritoneal ascites and solid prostate cancer tissue, resulted in the upregulation of PD-1 expression on TILs, followed by the destruction of CAFs. In conclusion, EnAd blood stability and systemic bioavailability make it a potential virus platform for targeted BiTE expression in tumors. This approach to trigger proinflammatory cell death and reverse TME-mediated immunosuppression may be required to transform uncompromising, stromal-rich carcinomas into immunotherapeutic targets (124).

Another evaluation of CAF-targeting through applying of the ICOVIR oncolytic adenovirus platform was conducted in order to explore the efficacy of OV-BiTE (125). The two studies conducted by Freedman et al. and de Sostoa et al. elucidate similar methodologies and concepts (124, 125). In contrast to the EnAd-FAP-BiTE research, the study conducted by de Sostoa et al. included immunodeficient mouse models instead of clinical samples in order to assess efficacy (125). The evaluation of T cell biological distribution and efficacy against tumors has been conducted *in vivo*. The interaction between FBiTE and CD3+ effector T cells, as well as FAP+ targeted cells, resulted in the activation of T cells, their subsequent proliferation, and the induction of cytotoxicity leading to the death of FAP-positive A549 tumor cell lines. In the Hu-SCID tumor models, the expression of FBiTE in OVs was found to augment the intratumoral retention and accumulation of T cells while concurrently reducing the level of FAP expression in the treated tumors. The anti-cancer beneficial effects of the FBiTE-armed OV exhibited a notable superiority over the unmodified viral strain (125). Taken together, the findings from these studies indicate that BiTE-armed OVs have the ability to selectively target malignant cells as well as the stroma associated with tumors, hence encouraging improved therapeutic effectiveness.

Scott et al. designed a research experiment wherein they successfully developed BiTE-armed Ad viruses as well as TriTE-armed Ad viruses. The study demonstrated the efficacy of these viruses in the eradication of TAMs in samples obtained from patients suffering from several malignancies such as melanoma, ovarian cancer, breast cancer and gastrointestinal cancers. In the present study, a comprehensive assortment of bi- and tri-valent T cell engagers was precisely constructed, with the primary objective of targeting CD3ϵ on T cells and CD206 or folate receptor β (FRβ) on M2-like macrophages. T-cell engagers were genetically integrated into the genome of EnAd, a viral vector, and subsequently evaluated for their oncolytic activity and secretion of BiTE in the presence of tumor cells. Overall, this study designed an oncolytic adenovirus, EnAd, to express TAM-targeting T cell engagers without affecting its oncolytic activity, developing a multi-prolonged therapeutic approach to target cancer cells and immunosuppressive TAMs. In summary, this study predicts that eliminating cancer-promoting TAMs, along with the immune-boosting effects of BiTEs and OVs, will offer a potent treatment strategy for overcoming obstacles to anti-tumor immunity in cancer patients (122).

The T lymphocytes, upon activation by the CD206 and FRβ-targeting BiTEs/TriTEs, demonstrate a preference for the selective elimination of M2-polarized autologous macrophages as opposed to M1-polarized autologous macrophages. A novel TriTE, possessing bivalent CD3ϵ binding, showed enhanced efficacy while maintaining its selectivity towards target cells. In contrast, a TriTE containing CD28 has induced non-specific activation of T cells. In immunosuppressive malignant ascites, the activation of endogenous T cells and the production of IFN-γ were observed upon exposure to both free and EnAd-encoded T cell engagers. This resulted in a notable expansion of T cell populations and a reduction in the presence of CD11b+CD64+ ascites macrophages. Remarkably, the macrophages that succeeded to survive demonstrated a notable elevation in the expression of M1 markers. This observation indicates a potential shift in the microenvironment towards a state of pro-inflammatory response (122). The results of this study suggest that there is significant potential in the field of viral vectors and BiTE/TriTE molecule engineering for the development of safer and more effective cancer immunotherapy. However, further investigation into the mechanisms underlying the OV-BiTE therapeutic approach is recommended.

The treatment landscape for recurrent or refractory (R/R) B-cell malignancies has been significantly impacted by the substantial advancements achieved in CD19-based immunotherapy in recent years (137, 138). Blinatumomab, a BiTE targeting CD19 and CD3, has received approval for use in the relapsed/refractory (R/R) B-cell precursor ALL. This approval is based on evidence gathered from the Phase III TOWER study, which demonstrated notable enhancements in overall survival and remission rates when compared to the conventional chemotherapy (139). Nevertheless, NHL patients who demonstrate extramedullary involvement may display greater resistance towards BiTE therapy, indicating a potential constraint in the ability of BiTEs to infiltrate the tumor sites. Additional limitations include the relatively brief half-life of blinatumomab, necessitating a continuous infusion spanning a duration of 6 to 8 weeks. This temporal constraint represents a significant challenge to its clinical application. Furthermore, a notable feedback is that a majority of patients who received this therapeutic agent experienced rate 3 or greater adverse events (139, 140). To address these problems, Wen et al. developed an oncolytic vaccinia virus (OVV) that encodes a CD19-specific BiTE (OVV-CD19BiTE). The findings indicate that the replication and oncolytic properties of OVV-CD19BiTE were comparable to those of its parental counterpart. The induction of activation and proliferation of human T cells, as well as the bystander effect of the virus, were observed upon exposure to supernatants derived from OVV-CD19BiTE-infected cells. The *in vivo* investigation demonstrated that OVV-CD19BiTE displayed selective replication within the tumor tissue, resulting in a notably augmented proportion of CD3, CD8, and naïve CD8 T subpopulations within the tumor, as compared to blinatumomab. Furthermore, it is of utmost significance to note that the administration of OVV-CD19BiTE, both *in vitro* and in animal models, exhibited remarkable efficacy in combating tumor growth when compared to the control group receiving control OVs or blinatumomab (128). This research presents compelling evidence regarding the therapeutic advantages of CD19-targeting BiTE expression through Oncolytic Vaccinia Virus. This novel OVV has the potential to overcome the limitations observed in current BiTE therapy, leading to significant therapeutic benefits in the management of B-cell lymphomas. Furthermore, it recommends the possibility of evaluating that therapeutic approach in clinical trials.

In several recent studies, the herpes simplex virus has been employed as an efficient vector for BiTEs. A study has revealed that the administration of oncolytic herpes simplex virus type 1 (HSV-1) has the ability to reprogram the TME with immunosuppressive characteristics into a state that is more proinflammatory. Specifically, it has been observed that the presence of oncolytic HSV-1 leads to a significant decrease in the population of anti-inflammatory macrophages in the TME (141). Furthermore, the administration of CD40L-expressing HSV-1 therapy demonstrated the ability to induce dendritic cell maturation and activate cytotoxic T cells. This therapeutic intervention substantially extended the lifespan of mice suffering from pancreatic ductal adenocarcinoma (PDAC) (142). The results of this study have strengthened the hypothesis of the effectiveness of oHSV-CD40L when combined with ICIs in targeting the PD-1/PD-L1 pathway for overcoming PDAC. Moreover, clinical trials are currently underway to study the potential of HSV Type 2 in treating a variety of solid cancers, including melanoma (**NCT03866525**). These studies provide evidence of the therapeutic potential of HSV in the treatment of various malignancies.

Khalique et al. conducted a study wherein they armed oncolytic herpes simplex virus-1 (oHSV-1) with PD-L1 BiTE. The objective was to evaluate the efficacy of this combination in delivering targeted cytotoxicity in unpurified cultures of malignant ascites obtained from diverse cancer patients. The findings of the study demonstrate that PD-L1 BiTE exhibits notable efficacy as an immunotherapy agent for killing PD-L1-positive tumor cells and macrophages, while concurrently preserving the integrity of T lymphocytes. Using an OV for the purpose of local expression of PD-L1 BiTE not only helps prevent the occurrence of systemic toxicities associated with ‘on-target off-tumor’ effects but also have the ability to overcome the TME immunosuppressive conditions (130). In another study Shiyu Liu et al. developed a murine OX40L BiTE-armed oHSV-1 (OV-mOX40L). The administration of OV-mOX40L resulted in the transformation of the immunosuppressive tumor immunological environment into a state of elevated activation, accompanied by the restructuring of the stromal matrix and stimulation of T cell response. The administration of OV-mOX40L demonstrated a significant increase in the lifespan of mice with pancreatic ductal PDAC, whether used as a monotherapy or in combination with complementary antibodies that exhibited synergistic effects (131). The results of this study offer significant evidence supporting the effectiveness of OV-mOX40L treatment. These results have the potential to make valuable contributions to the development of OV-mOX40L as a monotherapy or as part of a combination therapy for PDAC.

Jing Jin and colleagues performed a study in which they developed BiTEs targeting PD-L1 or CD19 (oHSV2-BiTEs-PD-L1 or oHSV2-mBiTEs-CD19). The findings of their study indicate that the oHSV2-BiTEs showed enhanced oncolytic potency both *in vitro* and *in vivo*. The oHSV2-BiTEs-PD-L1 construct has the ability to trigger oncolysis in tumor cells that have been infected. Additionally, it can stimulate PBMCs by releasing BiTEs-PD-L1, which leads to the PBMCs-mediated elimination of tumor cells that express PD-L1, irrespective of the level of PD-L1 expression. Furthermore, it has been shown that both oHSV2 and PBMCs have the ability to enhance the expression of PD-L1 on tumor cells. oHSV2-BiTEs-PD-L1 and oHSV2-mBiTEs-CD19 demonstrated an elevated oncolytic effect both *in vitro* and *in vivo* when compared to the control group, which involved the backbone virus oHSV2-GFP (132). The study’s findings indicate that the oHSV2, armed with BiTEs molecules, possesses the capability to transform T cells into potent tumor-killing cells, thereby enhancing the effectiveness of antitumor treatment. This suggests that it holds great potential as a potential therapy for future cancer clinical trials.

## 6 Combination therapy: CAR-T cells and OV-armed BITEs

Tumor antigen heterogeneity poses a significant challenge in the context of therapeutic interventions involving chimeric antigen receptor (CAR) T cells and bi- or Tri-specific T-cell engagers armed with OVs (104). In order to address this significant concern, two studies have been designed employing BiTE-OVs in combination with the adoptive transfer of CAR-T cells.

In a study published by Wing et al., it was demonstrated that CAR-T cells armed against FR-α effectively infiltrated tumors. However, these CAR T cells were unable to achieve robust responses, likely attributable to the existence of FR-α-negative malignant cells induced by tumor evasion. Through the combination of ICO15K-cBiTE AdV, which encodes an EGFR-targeting BiTE, with FR-α-specific CAR T cells, the objective of this study was to address the issue of tumor heterogeneity and the potential loss of tumor antigens. The findings revealed that Ad-BiTE-mediated oncolysis indicated a noteworthy enhancement in the activation and proliferation of CAR-T cells. Additionally, it led to an enhancement in cytokine production and cytotoxicity, thereby displaying a favorable safety profile *in vitro* when compared to CAR-T cell-armed EGFR. BiTEs are synthesized and released by cells that have been infected which have the ability to redirect CAR-T cells towards epidermal EGFR, even without the presence of FR-α. This redirection of CAR-T cells plays a crucial role in addressing the heterogeneity of tumors. The secretion of BiTE additionally directs CAR-negative, non-specific T cells that are present in CAR-T cell preparations towards cancer cells. The implementation of a combination methodology exhibited enhanced antitumor efficacy and long-term survival in mouse cancer models, in contrast to the monotherapies. This favorable outcome can be attributed to an enhanced activation of T-cells mediated by BiTE within the TME (126).

In these concept, Porter et al. applied an OV designed for simultaneously producing IL-12, an anti-programmed cell death ligand-1 (PD-L1) antibody, and a CD44 variant6-targeting BiTE, thereby creating a combined agent stated as CAdTrio (127). Given the significant expression of CD44v6 on tumor tissue and its absence in normal tissue, it is noteworthy that the administration of a CD44v6 antibody to patients suffering from cancers has been associated with reduced adverse effects (127, 143, 144). The CD44v6 BiTE, when expressed from CAdTrio, facilitated the cytotoxicity of HER2-specific CAR-T cells against various CD44v6+ cancer cell lines. Additionally, it resulted in a more expedited and prolonged treatment of disease in orthotopic HER2+ and HER2− CD44v6+ tumor cells. The administration of CAdTrio, in combination with HER2.CAR T cells, facilitated the achievement of dual targeting of two tumor antigens through the engagement of separate receptor classes (CAR and native receptor [TCR]), thereby enhancing therapeutic outcomes (127). In summary, the findings of this studies indicate that simultaneous administration of a BiTE-expressing OVand adoptive CAR-T cell therapy effectively addresses the fundamental drawbacks of CAR-T cells and BiTEs when used as monotherapy for solid tumors. These results provide compelling evidence to support the demand for further research of this combined approach in clinical trials.

## 7 Concluding remarks

The rapid advancements in molecular biotechnology have facilitated the development of innovative approaches for harnessing the immune system for the management of cancer. At now, several methodologies, such as adoptive cell treatments, monoclonal antibodies, checkpoint inhibitors, and OVs, are considered major advancements in the field of cancer treatment. These approaches have demonstrated the ability to deliver long-lasting and efficient clinical outcomes to cancer patients. Nevertheless, it is imperative to note that currently, the therapeutic advantages of immunotherapy are confined to a restricted subset of patients who undergo treatment. Solid tumors often possess a tumor microenvironment that is characterized by its ability to decrease the activity of T cells and facilitate tumor development. Furthermore, the emergence of novel immunotherapy treatments has given rise to the appearance of previously unobserved immunological adverse effects, such as cytokine storm and autoimmune disorders. Given these drawbacks, it is imperative to make additional modifications to these therapeutic procedures. In addition to novel immunotherapeutic approaches, it is imperative to enhance our knowledge of a patient’s immune contexts in order to improve patient benefits.

FDA and EuEU authorities have approved armed OVs, including T-VEC, in the treatment of patients diagnosed with advanced-stage melanoma. This approval has established armed OVs as a prominent example for the ongoing development of OVs. Regarding BiTEs, it is worth noting that blinatumomab, a dual-specific antibody targeting CD19 and CD3, has demonstrated enhanced efficacy in treating patients diagnosed with B cell lymphoma.

Due to the OVs and BiTEs/TriTEs limitations in solid tumor treatments, the use of BiTE- or TriTE-armed OVs poses an attractive and efficient approach for addressing this unresolved clinical requirement, especially when employed in combination with supplementary methods aimed at mitigating the immunosuppressive tumor microenvironment. These efforts are expected to result in the creation of innovative anti-cancer therapeutic approaches, such as enhanced T cell engagers. This particular goal is recognized as one of the most significant challenges in the field of cancer immunology. The OV-BiTE/TriTE methodology serves as a prime instance in this context. Based on the reliable rationale for science, numerous preclinical research have substantiated the proof-of-concept for this particular approach. Therefore, it is imperative for OV-BiTEs to exhibit both practicality and effectiveness in a clinical setting.

## Author contributions

AZ: Conceptualization, Investigation, Validation, Visualization, Writing – original draft, Writing – review & editing. MT: Writing – original draft. AG: Writing – original draft. FR: Writing – original draft. HE: Conceptualization, Supervision, Validation, Writing – review & editing.
